# Deep-Reinforcement-Learning-Based IoT Sensor Data Cleaning Framework for Enhanced Data Analytics

**DOI:** 10.3390/s23041791

**Published:** 2023-02-05

**Authors:** Alaelddin F. Y. Mohammed, Salman Md Sultan, Joohyung Lee, Sunhwan Lim

**Affiliations:** 1 School of Computing, Gachon University, Seongnam 13120, Republic of Korea; 2 European IT Solutions Institute, Dhaka 1216, Bangladesh; 3 Autonomous IoT Research Section, ETRI, Electronics and Telecommunications Research Institute, Daejeon 34129, Republic of Korea

**Keywords:** IoT, DQN, edge intelligence, data cleaning

## Abstract

The Internet of things (IoT) combines different sources of collected data which are processed and analyzed to support smart city applications. Machine learning and deep learning algorithms play a vital role in edge intelligence by minimizing the amount of irrelevant data collected from multiple sources to facilitate these smart city applications. However, the data collected by IoT sensors can often be noisy, redundant, and even empty, which can negatively impact the performance of these algorithms. To address this issue, it is essential to develop effective methods for detecting and eliminating irrelevant data to improve the performance of intelligent IoT applications. One approach to achieving this goal is using data cleaning techniques, which can help identify and remove noisy, redundant, or empty data from the collected sensor data. This paper proposes a deep reinforcement learning (deep RL) framework for IoT sensor data cleaning. The proposed system utilizes a deep Q-network (DQN) agent to classify sensor data into three categories: empty, garbage, and normal. The DQN agent receives input from three received signal strength (RSS) values, indicating the current and two previous sensor data points, and receives reward feedback based on its predicted actions. Our experiments demonstrate that the proposed system outperforms a common time-series-based fully connected neural network (FCDQN) solution, with an accuracy of around 96% after the exploration mode. The use of deep RL for IoT sensor data cleaning is significant because it has the potential to improve the performance of intelligent IoT applications by eliminating irrelevant and harmful data.

## 1. Introduction

In the time of the 4.0 industrial revolution, real-world things can be converged and implemented with the help of the Internet of things (IoT) [1]. Therefore, millions of devices with sensors and actuators are connected via wired or wireless channels for data transmission to operate the IoT. However, IoT devices generate a lot of data from connected devices with various modalities and qualities. It is predicted in [2] that the connected devices may generate approximately 79.4 zettabytes (ZBs) of real-time data. The IoT is a rapidly growing field that involves the connection of a wide range of devices and sensors to the internet, enabling the collection and analysis of data from these devices in real time. The IoT has the potential to revolutionize a wide range of industries, including healthcare, transportation, and agriculture, by providing organizations with insights into the operations and performance of their systems. However, the success of the IoT depends on the quality of the data being collected and analyzed. Due to the significant enhancement of IoT-based sensing data, it is crucial to maintain the quality of the data with a higher priority. To meet the IoT services for the users, the IoT utilizes three to five layers of architecture depending on different IoT applications [3,4,5]. In general, the five-layers architecture (i.e., sensing layer (SL), communication layer (CL), data processing layer (DPL), data management layer (DML), and application layer (AL)) is more used than the three-layer architecture due to its high data-quality-maintaining capability in any extensive IoT application [3].

Machine Learning (ML) is promising in the 4.0 industrial technology era, mainly utilized for handling big data environments efficiently. The data processing layer is considered the most crucial in ML-based IoT applications. A real-time IoT application’s ability to use memory more effectively depends on the data processing layer of IoT architecture. The likelihood of receiving undesired data from IoT sensors is very high.

Most IoT-based ML applications demand a clean data environment. However, different undesirable and noisy data could be generated by IoT sensors [6]. To overcome noisy data, some proposals were introduced in the literature. Three scopes were outlined in [7] for detecting dirty data from a large dataset to ensure data quality (i.e., what to detect, how to detect, and where to detect). Following that, the authors only focused on detecting the integrity constant, functional dependencies, and denial constraint types of errors in an extensive database. In the overview of their study, an ML technique called ActiveClean was introduced and employed for generating clean data intelligently. To improve data quality in any IoT application, [8] evaluated the data quality process under validity and completeness criteria. The validity indicated different constraints (e.g., data efficiency, statistical validity). On the other hand, the completeness criteria were studied by evaluating the degree to which data were allowed to be observed. While measuring the completeness criteria, [8] gave an example of insufficient data called “Null” data and described the consistency problem that must be considered to maintain data quality.

The authors in [9] proposed an IoT gateway framework to increase data quality efficiency. In their case, a framework that could detect outliers and missing data from a time-series-based dataset was employed. They utilized the Message Queuing Telemetry Transport (MQTT) protocol and a Docker container to employ the gateway. After detecting the noisy data, they applied an exponential smoother to overcome this data issue. However, the above works did not apply any intelligent technique to detect unwanted or dirty data to improve the IoT data quality. To specify the intelligent system for improving the data collection process, [10] developed a prediction system to detect noisy data in a real-time IoT environment. The study utilized the Kalman filter to predict the upcoming outliers from a real-time data stream to employ the prediction system. [6] proposed a decentralized system to efficiently control the data cleaning process. They applied an advanced decentralized-based deep learning method called federate learning to improve the data quality independently. They focused on edge-based systems to overcome the latency issue in any centralized system. As a result, their federated learning method cleaned the noisy data in a decentralized manner, which increased the system’s efficiency. Furthermore, [11] deployed an intelligent-agent-based system using deep reinforcement learning to select robust features for cleaning the collected data from multiple sources. Their algorithm improved data quality better than the traditional reinforcement learning algorithm. However, there is room to improve the data quality using ML techniques.

This paper proposes a deep reinforcement learning (deep RL)-based ML technique for handling unnecessary data from IoT sensors. The proposed method, called recurrent-neural-network-based long short-term memory Q-network (RLQN), deploys a deep Q-network (DQN) to take appropriate action after detecting different types of unnecessary data. In our proposed DQN system, the input state consists of three received signal strength (RSS) values, indicating the current RSS and the last two RSS sensor data. In addition, the garbage status (i.e., whether the current RSS value is garbage or not) is also included in the input state for the DQN. We evaluate our proposed RLQN using RSS sensor data and compare it to a time-series-based fully connected neural network solution called dense Q-network (FCDQN). The main activity of our proposed RLQN is to decide the action that achieves the best Q-values among many predicted Q-values. The proposed DQN agent receives reward feedback based on the predicted action it takes. Our results show that the proposed model is far superior to the existing FCDQN in terms of cleaning garbage and empty data, which helps to improve the data analytics process.

Overall, the proposed RLQN is a promising approach for handling unnecessary data from IoT sensors in a real time. One of the key advantages of the proposed RLQN is its ability to handle a large number of inputs and make decisions in real time. This is especially important for IoT applications, where hundreds or thousands of sensors may send data simultaneously. By using the proposed RLQN to process these data and identify unnecessary or irrelevant information, the proposed RLQN can significantly improve the efficiency and accuracy of data analytics. In addition to its real-time decision-making capabilities, the proposed RLQN is also highly adaptable and can be easily fine-tuned to different environments and situations. This makes it a versatile solution for many IoT applications and can help organizations better manage and analyze their data.

The remainder of this paper is organized as follows. In Section 2, we introduce the related work and provide an overview of previous research in this field. In Section 3, we present the system preliminaries, including a detailed description of the proposed RLQN and its underlying algorithms. Section 4 outlines the simulation setup and presents the results of our experiments, including a detailed analysis and comparison with other methods. Finally, in Section 5, we provide a summary of our findings and discuss potential future directions for this research.

## 2. Related Work

Data cleaning is a crucial step in the process of collecting and analyzing data from IoT devices. It is important because it helps to ensure the accuracy and quality of the data, which is essential for making informed decisions and taking appropriate actions. Several studies have highlighted the importance of data cleaning in IoT. The methods used for data cleaning can be broadly divided into three categories: user-based, rule-based, and machine-learning-based.

User-based cleaning is the process of identifying and correcting errors, inconsistencies, and missing values in data by manually reviewing the data and making corrections by hand. For instance, in [12], users can manually clean the data. Data cleaning is suitable for small to moderate datasets but can be time-consuming and prone to human errors. Moreover, it could be a tedious task when working with large datasets.

Rule-based methods rely on predefined rules and heuristics to clean the data. These methods are simple to implement and understand but may not be able to adapt to changes in data distribution. Examples of rule-based data cleaning methods for IoT include:

- Data validation involves checking the integrity and accuracy of data. Most ML applications demand a clean data environment and meet specific criteria, such as being within a certain range or having a specific format. Any piece of data that does not meet these criteria are flagged or removed. This method was used in [13] to create a system for validating encrypted data that allowed the edge device to process and clean the encrypted data before they were uploaded.
- Data deduplication involves removing duplicate data from the dataset, as IoT devices may collect the same data multiple times. Authors in [14] utilized this method and proposed a six-step framework for removing duplicates in records. They showed how the framework worked using a simple example from a research institution’s information systems, including publications and research projects.
- Data normalization involves converting data into a consistent format, such as converting measurements from different units into a single unit. [15] is an example of this approach.

Machine-learning-based methods can be used to automatically identify and correct errors and inconsistencies in the data. These methods are more flexible and able to adapt to changes in the data but may be more complex to implement and require more computational resources. Examples of machine-learning-based data cleaning methods for IoT include:

- Clustering: This technique involves grouping similar data points together and can be used to identify and correct errors and inconsistencies in the data. For instance, [16] proposed an algorithm for removing replicated records that were clustered-based, and the effectiveness of data cleaning methods was evaluated.
- Anomaly detection: This technique involves identifying data points that deviate from the norm and can be used to identify and correct errors and outliers in the data. The work in [17,18,19] is an example of anomaly detection, and in [20], the authors conducted a comprehensive survey. They provided context on the difficulties that may arise when using anomaly detection methods on IoT data and presented illustrations of IoT anomaly detection applications that had been previously reported in the scholarly literature.
- Deep learning: This technique involves using neural networks to automatically identify and correct errors and inconsistencies in the data. Deep learning can be useful in analyzing unstructured data generated by IoT devices, such as images and audio. It can be used for image classification, speech recognition, and natural language processing tasks. Detailed information is presented in [21] for this method.
- Reinforcement learning: This technique can be used to optimize the performance of IoT devices by learning from their interactions with the environment and adjusting their behavior accordingly. It can be used to optimize energy consumption, optimize communication protocols, and learn how to avoid errors in IoT devices. Work in [11] is an example of this technique for data cleaning.

The survey in [22] is exceptional; it examined current methods for choosing, optimizing, and updating models in the field of automated ML. This was done to find the most suitable solutions for each stage of using ML algorithms for data analysis in the IoT and present a summary of it. Furthermore, the authors in [21,23] conducted a survey to examine processing techniques for data in the IoT context. The survey examined current research on data processing and provided background information on the topic. Additionally, literature reviews of recent advanced research on processing techniques were presented. It is important to note here that the choice of method and technique depends on the specific characteristics of the data and the requirements of the downstream task.

One of the main advantages of using a deep RL framework for IoT data cleaning is that it can allow the agent to learn from experience and improve its performance over time. This is because the agent is able to learn from the consequences of its actions, rather than being explicitly told what to do. This can be particularly useful for data cleaning tasks, as it can allow the agent to adapt to different types of data and learn how to handle noisy or missing data. In addition, deep RL frameworks can handle high-dimensional and complex environments, which makes them well-suited for dealing with large datasets such as those typically encountered in IoT applications.

One limitation is that most of the current research has focused on simulated environments, rather than real-world IoT systems. This means that the results of these studies may not necessarily generalize to real-world scenarios, and more research is needed to understand how well deep-RL-based approaches can perform in more complex and dynamic environments. Another limitation is that most of the current research has focused on single-agent systems, rather than multiagent systems. In real-world IoT systems, there may be multiple sensors and devices that are interacting with each other and the environment. This means that more research is needed to understand how deep-RL-based approaches can be extended to handle multiagent systems and the challenges that this brings.

## 3. System Model

**Figure 1:**
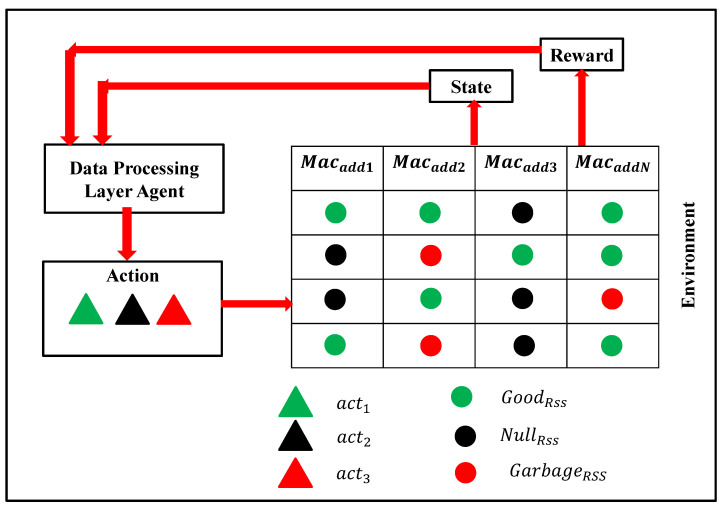
Deep reinforcement learning agent for tracking missing and garbage data.

Figure 1 illustrates the system overview of our proposed RLQN, where five different terminologies appear according to the concept of deep RL. Here, the agent of our deployed method is placed onto the data processing layer of the IoT architecture, where it decides an appropriate action based on a particular state. Note that the state is determined from a real-time environment.

The environment contains multiple sensor data, which are denoted by circles. In this case, we considered RSS-based sensor data which appeared under different media access control (MAC) addresses or access points. The environment is the key and essential concept in a DQN-based deep RL method because the agent constantly interacts with a particular environment for better performance. The interaction between the agent and environment generally occurs after selecting a specific action. Further, the environment helps to provide feedback to evaluate the agent’s action. The evaluated feedback (reward) plays a significant role in improving the agent’s performance. After getting the reward, the agent jumps to the next state from the environment to decide the following possible action using the epsilon-greedy method.

There are three types of sensor data (good, null, and garbage) received from sensing during data collection. In Figure 1, the good, null, and garbage data are represented by green, black, and red circles, respectively. Based on these different characteristics of the RSS-based state, our proposed agent takes an appropriate action which is further evaluated by providing a reward when interacting with the environment.

### 3.1. State Space

According to our presumption, unwanted data can be collected by the IoT sensor anytime due to noisy interactions or other vulnerable issues. As a result, it is imperative to deal with the properties of unwanted sensor data in the state space. In our proposed state space, we store three RSS values, where the first RSS represents the current RSS value ($RSS_{current}$), the next two RSS values are assumed to be the previous two RSS values ($RSS_{P_{1}}$, $RSS_{P_{2}}$), and one Boolean value represents the status of garbage value. The main reason for choosing the current RSS in the state space is to not identify the missing RSS data from the sensor. The $RSS_{C}$ can contain zero if there are any missing RSS values; otherwise, it can contain the actual RSS value received.

Furthermore, to replace the missing and garbage data, surround data (e.g., $RSS_{P_{1}}$, $RSS_{P_{2}}$) are also required along with the current RSS (i.e., $RSS_{C}$) value. Usually, the $RSS_{P_{1}}$ and $RSS_{P_{2}}$ would contain the RSS values of the last two data points unless there were no preceding data. Thus, in such cases, the value of $RSS_{P_{1}}$ would be minus one, whereas $RSS_{P_{2}}$ would be minus two for the state, or only the value $RSS_{P_{2}}$ would be minus one for the after state.

**Figure 2:**
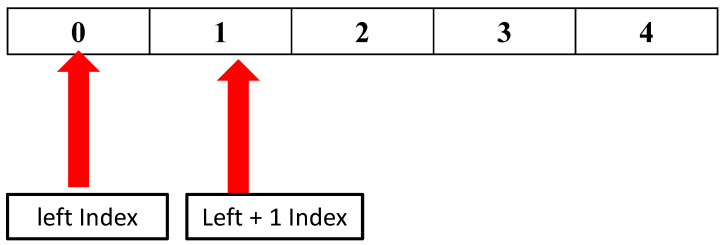
State space representation.

Figure 2 represents the state as an example, where we can observe that if the RSS value appears from the left index, the value of $RSS_{C}$ exists in this index. As there is no preceding data before the left index, the $RSS_{P_{1}}$ and $RSS_{P_{2}}$ are minus one and minus two, respectively. If the current RSS exists next to the left index, then the values of $RSS_{P_{1}}$ and $RSS_{P_{2}}$ are the values of the left index and minus one, respectively. After choosing these three RSS values, it is also essential to identify whether the RSS value is garbage or not; thus, we need to maintain a flag to keep track of it.

The state space in our proposed RLQN model is $s_{t}$ = {$RSS_{C}$, $RSS_{P_{1}}$, $RSS_{P_{2}}$, $flag_{G}$}. Table 1 explains these state space variables. Note that the value of the state space changes at each time step (*t*) during the data collection from the sensors.

### 3.2. Action Space

After receiving the state at a particular time from the state space, the proposed RLQN agent needs to take action from the designed action option. As the value of the state space is based on good RSS, garbage RSS, and null RSS, our proposed agent takes a specific action depending on the status of the RSS value in the state space.

Furthermore, due to noise and other environmental factors, some missing (null) or distorted (garbage) data are inevitable during data collection from sensors. These data types should not be stored on servers since they can significantly degrade performance. To prevent storing null and garbage data, our suggested approach seeks to recognize them. Therefore, the action space for this model is assumed to be limited from zero to two. The action is zero for identifying null or missing data, one for garbage data, and two for the rest of the data received.

**Figure 3:**
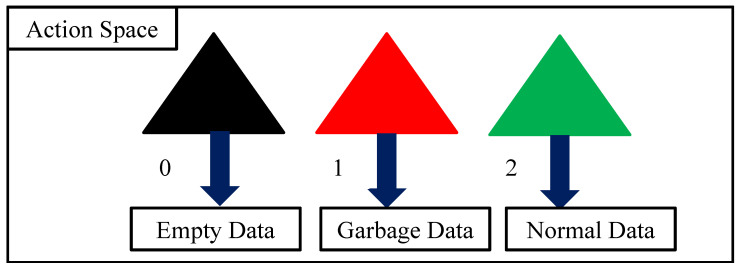
Possible three action spaces ($A_{P_{1}},A_{P_{2}},A_{P_{3}}$) of the proposed model.

Following that, the action space in this model can be represented as $A_{P_{K}}$ = { $A_{P_{1}},A_{P_{2}},A_{P_{3}}$}, where *K* is the number of data types, and *K* = 3 in our work. Note that we assumed different index numbers 0, 1, 2 for $A_{P_{1}}$,$A_{P_{2}}$, $A_{P_{3}}$, respectively. Figure 3 illustrates different types of action ($A_{P_{K}}$) that the proposed model can choose based on a state condition.

The criteria for selecting the course of action are as follows:

$$act_{scene}=\left\{\begin{array}{cc} 0, & \mathrm{if}\;(RSS_{C}=0);\\ 1, & \mathrm{if}\;(RSS_{C}<-100);\\ 2, & \mathrm{otherwise}; \end{array}\right.$$

### 3.3. Reward Space

The proposed method determines the agent’s performance by formulating a binary reward scheme. The primary benefit of binary rewards is their ease of estimation and absence of computational complexity. The reward also assists in evaluating the agent’s action to reach a decision very efficiently. As in (1), the reward is received by the proposed RLQN as a response at time *t*.

(1) $$r_{t}=\left\{\begin{array}{cc} One, & \mathrm{if}\;A_{P_{k}}\;=\;act_{scene}\\ Zero, & \mathrm{otherwise}, \end{array}\right.$$

where $A_{P_{k}}$ is the possible three action spaces as mentioned in Figure 1.

### 3.4. Agent Gaining and Storing Experience in an Experience Replay Memory

In our proposed RLQN-based system, acquiring experience by interacting with the mentioned environment is one of the ordinary and essential tasks to perform in an optimal manner. Initially, our designed DQN agent has no idea or experience with the system environment; thus, the agent randomly decides on a particular action using the epsilon parameter. Following that, the agent starts providing the best action, $best_{act}$, according to the greedy-based exploiting procedure as in (2).

(2) $$best_{act}=\arg\max\left(agent\left(state\right)\right).$$

**Figure 4:**
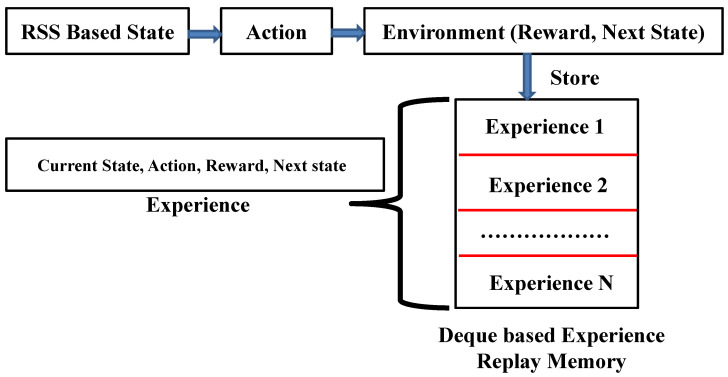
Deque-based experience replay memory.

To remember each situation automatically, the system stores the agent’s experience in a deque-based memory called experienced replay memory at a particular time step (t). Note that the experience of our proposed agent indicates the collection of the current state, action, reward, and next state together at each time step. This means the system saves these four values as a tuple into a single deque-based experience replay memory as presented in Figure 4.

### 3.5. Minibatch Exploring during Training

After storing each experience in the experience replay memory, the proposed system starts training immediately. To achieve a better outcome from the proposed agent, the system does not train all the experiences together at a particular time. Instead, the proposed system utilizes a minibatch that assists in sampling a set of data (i.e., 16 experiences) during the training period. However, sampled experiences can be correlated with each other if the minibatch technique does not collect experiences randomly. A higher correlation between the experiences can enhance the training complexity, which is one of the reasons for getting bad output from the system. To overcome this issue, we randomly applied the minibatch technique to sample a set of experiences from the experience replay memory and continue the training at each time step.

### 3.6. Proposed Q-network for Greedy Action Prediction during Training

**Figure 5:**
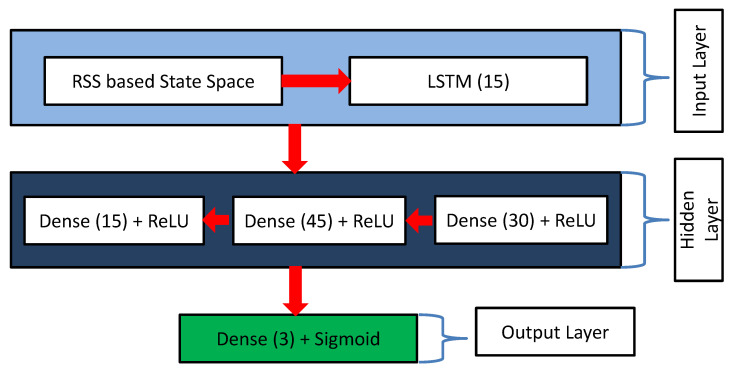
Proposed RLQN.

Designing a Q-network using a deep learning approach is extremely crucial for predicting a better action from the RSS-based state as input. In this study, we utilized RLQN, where LSTM was deployed as a Q-network to identify the type of sensor data in a particular state. The main reason for using LSTM to deploy our proposed Q-network was that LSTM can predict an action in any time-series-based environment. Our system collected RSS data from sections in real time; thus, LSTM was one of the best choices as a Q-network. Furthermore, we combined a fully connected dense network with our proposed RLQN to make the overall network robust. Figure 5 represents the proposed RLQN for our designed system.

Moreover, to enhance the system’s overall performance, we utilized another RLQN with the same structure called target-RLQN in this case. A primary objective of the DQN was to provide optimal action by optimizing the loss (Loss(θ)) as much as possible between predicted Q-values (*Q*) and target Q-values ($Q^{\prime }$). Our two RLQNs were responsible for giving the predicted and target Q-values results. The estimation of the loss (Loss(θ)) was maintained according to (3).

(3) $$\;Loss\left(\theta \right)={\left(Q^{\prime }-Q\right)}^{2}.$$

Note that, the details of *Q* and $Q^{\prime }$ are given in (4) and (5), respectively, where γ represents the discount factor ∈[0,1]. The overall algorithm of our proposed system is given in Algorithm 1.

(4) $$\;Q=Q(s_{t},act_{t};\theta ).$$

(5) $$\;Q^{\prime }=r_{t}+\gamma \max\left(Q\left(s_{t+1},act_{t+1};\theta^{\prime }\right)\right).$$

**Algorithm 1:** Proposed RLQN algorithm.

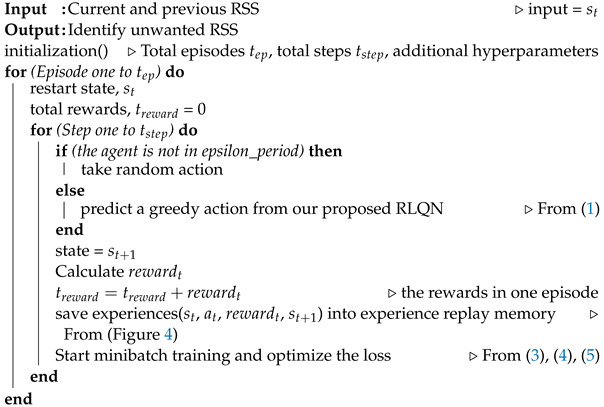



## 4. Result and Analysis

The proposed model was deployed on the TensorFlow 2.2.0 API under python 3.10 along with the Keras library. We evaluated our proposed DQN performance total rewards in one episode and unwanted data detection accuracy.

### 4.1. Proposed Environment for Evaluation

**Figure 6:**
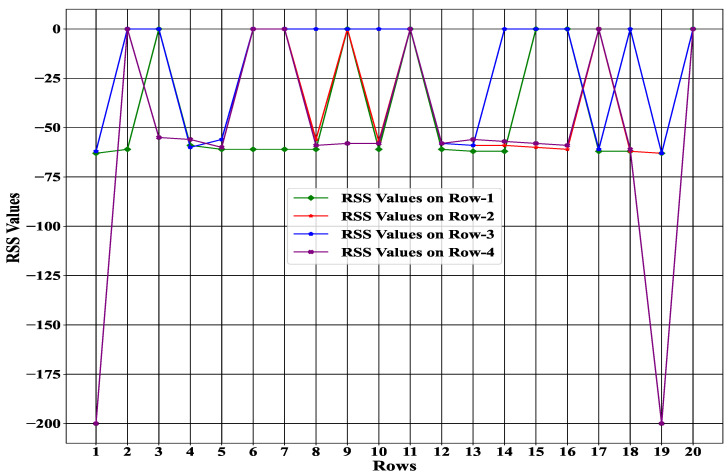
Example of some rows with 20 RSS values.

The environment is one of the essential factors in our proposed system to enhance the interactive performance of the agent. In our proposed environment, we assumed a grid-based architecture with a size of 20×20. Each index of the grid represented an RSS value that was collected from the sensor. As a result, we collected 400 RSS values in total, where each row had 20 RSS values. In other words, our proposed system evaluated 400 different state spaces, which could be achieved from the environment. Note that with the proposed state space, the proposed experience-driven method was efficient for inferring the solution after training. Figure 6 represents some row examples with 20 RSS values where all RSS data (good, null, and garbage) are available.

**Figure 7:**
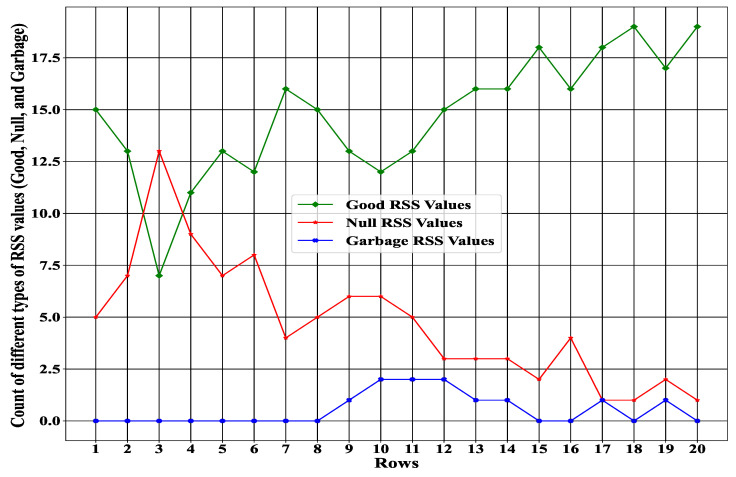
Total numbers of different RSS values.

Furthermore, Figure 7 illustrates the total number of different types of RSS values (good, null, garbage) in a specific row. It can be observed from the Figure that the system never achieved the RSS value from the sensor with 100% accuracy. During data collection, some data could be null or garbage. Due to this kind of unwanted RSS data, we needed to check the status of each RSS value based on our state space to improve the data collection process efficiently. Moreover, from Figure 6 and Figure 7, it can be seen that the variation of the RSS value was not fixed, and the status of the RSS value changed frequently over time. As a result, it was essential to take a particular action by considering our proposed state space (environment) at each time instead of taking the whole space.

### 4.2. Training Hyperparameters

To design the RLQN, we initialized some hyperparameters (e.g., learning rate, number of episodes, and so forth) by a trial-and-error strategy during the training period. These hyperparameters controlled the characteristics of the Q-network to provide the best action. Table 2 describes the training hyperparameters of the proposed DQN in detail.

### 4.3. Total Rewards (Each Episode)

**Figure 8:**
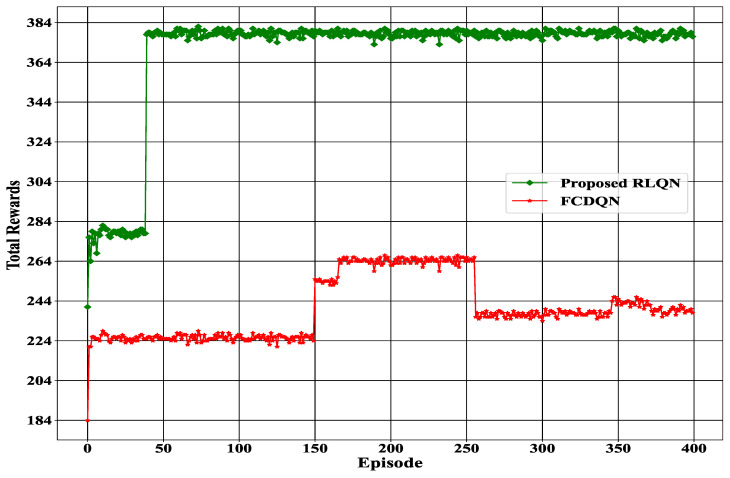
Total rewards for each episode.

Estimating total rewards in a particular episode was essential to properly evaluate the action of our proposed DQN agent. Figure 8 represents the total rewards for each episode. Note that we ran our model for 400 episodes because our environment had 400 RSS values.

As can be seen from Figure 8, both methods were able to achieve total rewards of more than 150 from the beginning and throughout the entire experiment. However, it can be noted that the total rewards achieved by FCDQN (184) were significantly lower than that of the proposed RLQN (241) at the start of the experiment. Furthermore, it can be observed that the proposed system experienced a significant increase in total rewards after 45 episodes, whereas the FCDQN experienced this increase only after 150 episodes, which was a much slower rate of improvement than that of the proposed method. Additionally, from the point of the increase in total rewards, the proposed Q-network was able to maintain a consistent level of rewards until the final episode. On the other hand, the FCDQN was able to maintain a consistent level of rewards for only a short period of time between 150 to 252 episodes, after which it again decreased in an inconsistent manner. This is evidence that the proposed system was able to produce better results than the FCDQN.

### 4.4. Detection Accuracy

**Figure 9:**
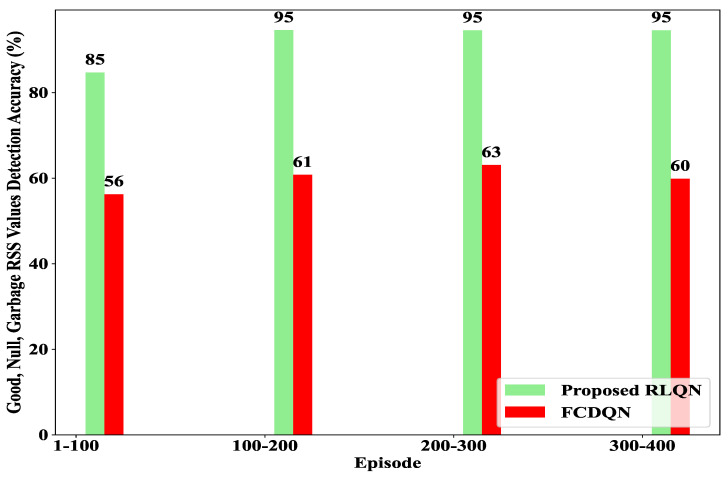
Detection accuracy for each episode interval.

The primary objective of the proposed system was to distinguish between different types of data, such as “Good”, “Null”, and “Garbage”, during the data collection phase in order to decrease the amount of unwanted data (i.e., “Null” and “Garbage”). Figure 9 illustrates the detection accuracy for identifying “Good”, “Null”, and “Garbage” RSS values until 400 episodes.

From this figure, it can be observed that the proposed method consistently achieved a higher accuracy at each episode interval. Additionally, it can be seen that the proposed system consistently attained an accuracy greater than 80% at each interval of the episodes, while the FCQN was unable to achieve an accuracy above 65%. It is worth noting that initially, the accuracy of the proposed method was lower than 90% due to the agent being in exploration mode during the first interval, but this increased to nearly 96% after the exploration period, as the agent started taking actions greedily. In contrast, the FCDQN never reached an accuracy of 70% by the final interval. Therefore, it can be concluded that the proposed system was highly robust in effectively identifying and removing “Null” and “Garbage” RSS data.

### 4.5. Improving Number of Good RSS

**Figure 10:**
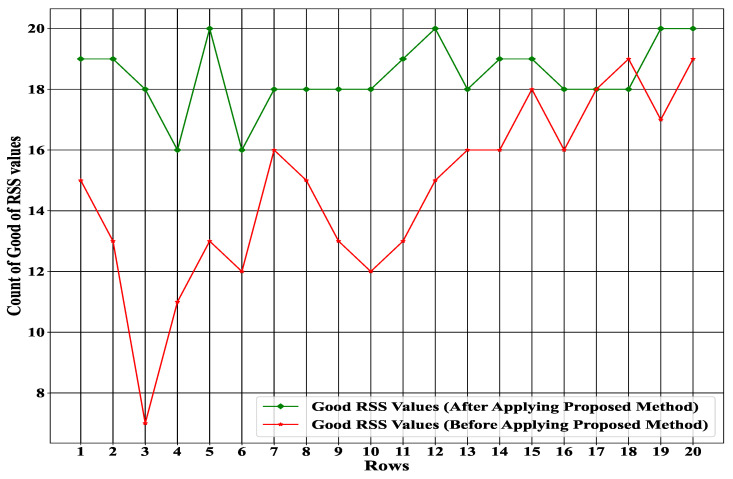
Improving the number of good RSS values before and after applying the proposed method.

As we observed from Figure 9, our proposed RLQN detection accuracy was much better than that of the FCDQN. To validate how much the counting of good RSS values can be improved after applying our proposed method, Figure 10 shows a transparent comparison of before and after applying our proposed method. Figure 10 demonstrates that the number of good RSS values increased significantly after applying the proposed method. Initially, the counting ratio fluctuated, but the outcome consistently increased until the final rows. Figure 10 proves that the proposed method can handle unwanted RSS values (null, garbage) to enhance the number of good RSS values. The larger number of good RSS values indicate the data quality was appropriately achieved using our proposed method.

Table 3 presents a summary comparison of various metrics between the proposed RLQN and FCDQN. From the table, it can be observed that the proposed RLQN method outperformed the FCDQN in terms of detection accuracy and total rewards, as the values for minimum, maximum, and average results were higher for the proposed RLQN method in all instances. This is important to note as the detection accuracy and total rewards are key metrics for evaluating the performance of the proposed system in this context. It provides a clear indication that the proposed RLQN method was more effective in identifying and removing unwanted data. Additionally, it is noteworthy that this comparison was made within the context of 400 episodes, highlighting the robustness of the proposed method over a prolonged period of time.

## 5. Conclusions

This paper proposed a deep RL IoT data cleaning framework to improve data analytics and handle unnecessary data from IoT sensors. The main objective of the proposed system was to identify and eliminate both null and garbage data, while preserving good data. To achieve this, we deployed a deep Q-network (DQN) to take appropriate action after detecting empty, garbage, and normal data. We evaluated our proposed framework using real-time RSS sensor data, and the results were compared against a common fully connected dense Q-network (FCDQN) solution. The results showed that the proposed solution achieved an accuracy of around 96% after the exploration mode.

One of the key advantages of the proposed framework is its ability to handle a large number of inputs and make decisions in real time. This is especially important for IoT applications, where there may be hundreds or thousands of sensors sending data simultaneously. By using the DQN to process these data and identify unnecessary or irrelevant information, the proposed framework can significantly improve the efficiency and accuracy of data analytics. In addition to its real-time decision-making capabilities, the proposed framework is also highly adaptable and can be easily fine-tuned to different environments and situations. This makes it a versatile solution for a wide range of IoT applications and can help organizations to better manage and analyze their data.

The proposed deep RL IoT data cleaning framework is a powerful tool for handling unnecessary data from IoT sensors and can greatly improve the efficiency and accuracy of data analytics in a variety of settings. In future work, we plan to further optimize and refine the proposed framework and to explore its potential for use in other domains and applications.

## Figures and Tables

**Table 1:** State space description.

Symbols	Description
$RSS_{C}$	Current RSS value
$RSS_{P_{1}}$	RSS value before the current RSS value $RSS_{C}$
$RSS_{P_{2}}$	RSS value before $RSS_{P_{1}}$
$flag_{G}$	Whether the current RSS value $RSS_{C}$ represents garbage data or not

**Table 2:** Training hyperparameters.

Hyperparameter	Value
Optimizer	Adam
Loss	Categorical cross-entropy
Batch Size	32
Experience replay memory size (*E*)	1000
Learning rate (*∂*)	0.0001
Factor of discount (γ)	0.7
The maximum epsilon	one
The minimum epsilon	0.001
The decay of the epsilon	0.995

**Table 3:** Summary comparison of the experimental results between the proposed RLQN and FCDQN within 400 episodes.

Metrics	Proposed RLQN	FCDQN
Minimum total rewards	241	184
Maximum total rewards	382	267
Average total rewards	368.48	240.03
Minimum detection accuracy	60.25%	46.0%
Maximum detection accuracy	95.5%	66.75%
Average detection accuracy	92.5%	60%

## References

[1] Makkar A., Garg S., Kumar N., Hossain M.S., Ghoneim A., Alrashoud M. (2020). An efficient spam detection technique for IoT devices using machine learning. IEEE Trans. Ind. Inform..

[2] Zhang L., Jeong D., Lee S. (2021). Data Quality Management in the Internet of Things. Sensors.

[3] Sethi P., Sarangi S.R. (2017). Internet of things: Architectures, protocols, and applications. J. Electr. Comput. Eng..

[4] Mashal I., Alsaryrah O., Chung T.Y., Yang C.Z., Kuo W.H., Agrawal D.P. (2015). Choices for interaction with things on Internet and underlying issues. Ad Hoc Netw..

[5] Wu M., Lu T.J., Ling F.Y., Sun J., Du H.Y. Research on the architecture of Internet of Things. Proceedings of the 2010 3rd international conference on advanced computer theory and engineering (ICACTE).

[6] Ma L., Pei Q., Zhou L., Zhu H., Wang L., Ji Y. (2020). Federated data cleaning: Collaborative and privacy-preserving data cleaning for edge intelligence. IEEE Internet Things J..

[7] Chu X., Ilyas I.F., Krishnan S., Wang J. Data cleaning: Overview and emerging challenges. Proceedings of the 2016 International Conference on Management of Data.

[8] Song S., Zhang A. IoT data quality. Proceedings of the 29th ACM International Conference on Information & Knowledge Management.

[9] Hallström F., Adolfsson D. (2021). Data Cleaning Extension on IoT Gateway: An Extended ThingsBoard Gateway. Ph.D. Thesis.

[10] Gudivada V., Apon A., Ding J. (2017). Data quality considerations for big data and machine learning: Going beyond data cleaning and transformations. Int. J. Adv. Softw..

[11] Wang Q., Guo Y., Yu L., Chen X., Li P. (2020). Deep Q-network-based feature selection for multisourced data cleaning. IEEE Internet Things J..

[12] Musleh M., Ouzzani M., Tang N., Doan A. Coclean: Collaborative data cleaning. Proceedings of the 2020 ACM SIGMOD International Conference on Management of Data.

[13] Xu L., Yuan X., Zhou Z., Wang C., Xu C. (2021). Towards Efficient Cryptographic Data Validation Service in Edge Computing.

[14] Azeroual O., Jha M., Nikiforova A., Sha K., Alsmirat M., Jha S. (2022). A Record Linkage-Based Data Deduplication Framework with DataCleaner Extension. Multimodal Technol. Interact..

[15] Fekade B., Maksymyuk T., Kyryk M., Jo M. (2017). Probabilistic recovery of incomplete sensed data in IoT. IEEE Internet Things J..

[16] Diachok R., Klym H. (2022). Data Cleaning method in wireless sensor-based on Intelligence Technology. Meas. Equip. Metrol..

[17] Singhal D., Meena J. Anomaly Detection in IoT network Using Deep Neural Networks. Proceedings of the 2021 IEEE 4th International Conference on Computing, Power and Communication Technologies (GUCON).

[18] Fahim M., Sillitti A. (2019). Anomaly detection, analysis and prediction techniques in iot environment: A systematic literature review. IEEE Access.

[19] Liu Y., Pang Z., Karlsson M., Gong S. (2020). Anomaly detection based on machine learning in IoT-based vertical plant wall for indoor climate control. Build. Environ..

[20] Cook A.A., Mısırlı G., Fan Z. (2019). Anomaly detection for IoT time-series data: A survey. IEEE Internet Things J..

[21] Jane V. (2021). Survey on iot data preprocessing. Turkish J. Comput. Math. Educ. (TURCOMAT).

[22] Yang L., Shami A. (2022). IoT data analytics in dynamic environments: From an automated machine learning perspective. Eng. Appl. Artif. Intell..

[23] Teh H.Y., Kempa-Liehr A.W., Wang K.I.K. (2020). Sensor data quality: A systematic review. J. Big Data.

